# Supplementary material for: Mental Health Interventions among Adolescents in India: A Scoping Review
Source: Healthcare (Basel). 2022 Feb 10;10(2):337. doi: 10.3390/healthcare10020337 (PMC8871588; doi:10.3390/healthcare10020337)
Supplement: Supplementary file 1 [file healthcare-10-00337-s001.zip › healthcare-1560771-supplementary.pdf]

healthcare

MDPI

Table S1. Joanna Brigg Institute Checklist for Quasi-experimental studies.

Checklist for Quasi-experimental studies [24]							
**Criteria**	**Singhal et al 2014 [26]**	**Sarkar et al 2017 [27]**	**Srikala et al 2010 [28]**	**Das et al. 2010 [29]**	**Singhal et al 2018 [32]**	**Balaji et al. 2011 [35]**	**Azeez 2015 [34]**
Is it clear in the study what is the "cause" and what is the "effect" (i.e., there is no confusion about which variable comes first)?	Yes	Yes	Yes	Yes	Yes	Yes	Yes
Were the participants included in any comparisons similar?	Yes	Yes	Yes	Yes	Yes	Yes	Yes
Were the participants included in any comparisons receiving similar treatment/care, other than the exposure or intervention of interest?	Yes	Yes	Yes	Yes	Yes	No	Yes
Was there a control group?	Yes	Yes	Yes	No	Yes	Yes	No
Were there multiple measurements of the outcome both pre and post the intervention/exposure?	Yes	Yes	Yes	Yes	Yes	Yes	Yes
Was follow up complete and if not, were differences between groups in terms of their follow up adequately described and analysed?	Yes	Yes	Not Given	Not Given	Yes	Yes	Yes
Were the outcomes of participants included in any comparisons measured in the same way?	Yes	Yes	Yes	Yes	Yes	Yes	Yes
Were outcomes measured in a reliable way?	Yes	Yes	Yes	Yes	Yes	Yes	Yes
Was appropriate statistical analysis used?	Yes	Yes	Yes	Yes	Yes	Yes	Yes

Table S2. Joanna Brigg institute checklist for Randomized control trials.

Checklist for Randomized Controlled Trials [24]		
	**Leventhal et al 2015 [30]**	**Shinde et al 2020 [31]**
Was true randomization used for assignment of participants to treatment groups?	Yes	Yes
Was allocation to treatment groups concealed?	No	Yes
Were treatment groups similar at the baseline?	Yes	Yes
Were participants blind to treatment assignment?	NG	Yes
Were those delivering treatment blind to treatment assignment?	No	Yes
Were outcomes assessors blind to treatment assignment?	No	Yes
Were treatment groups treated identically other than the intervention of interest?	Yes	Yes
Was follow up complete and if not, were differences between groups in terms of their follow up adequately described and analyzed?	Yes	Yes
Were participants analyzed in the groups to which they were randomized?	Yes	Yes
Were outcomes measured in the same way for treatment groups?	Yes	Yes
Were outcomes measured in a reliable way?	Yes	Yes
Was appropriate statistical analysis used?	Yes	Yes
Was the trial design appropriate, and any deviations from the standard RCT design (individual randomization, parallel groups) accounted for in the conduct and analysis of the trial?	Yes	Yes

Table S3. Joanna Brigg institute checklist for Qualitative studies.

**Checklist for Qualitative Research [25]**	
	**Chandra et al 2014 [36]**
Is there congruity between the stated philosophical perspective and the research methodology?	Yes
Is there congruity between the research methodology and the research question or objectives?	Yes
Is there congruity between the research methodology and the methods used to collect data?	Yes
Is there congruity between the research methodology and the representation and analysis of data?	Yes
Is there congruity between the research methodology and the interpretation of results?	Yes
Is there a statement locating the researcher culturally or theoretically?	Yes
Is the influence of the researcher on the research, and vice- versa, addressed?	Yes
Are participants, and their voices, adequately represented?	Yes
Is the research ethical according to current criteria or, for recent studies, and is there evidence of ethical approval by an appropriate body?	Yes
Do the conclusions drawn in the research report flow from the analysis, or interpretation, of the data?	Yes

Table S4. Joanna Brigg institute checklist for Cohort studies.

**Checklist for Cohort Studies [24]**	
	**Michelson et al 2019 [33]**
Were the two groups similar and recruited from the same population?	Yes
Were the exposures measured similarly to assign people to both exposed and unexposed groups?	Yes
Was the exposure measured in a valid and reliable way?	Yes
Were confounding factors identified?	No
Were strategies to deal with confounding factors stated?	No
Were the groups/participants free of the outcome at the start of the study (or at the moment of exposure)?	Yes
Were the outcomes measured in a valid and reliable way?	Yes
Was the follow up time reported and sufficient to be long enough for outcomes to occur?	Yes
Was follow up complete, and if not, were the reasons to loss to follow up described and explored?	Yes
Were strategies to address incomplete follow up utilized?	No
Was appropriate statistical analysis used?	Yes

healthcare MDPI

Table S5. Detailed characteristics of the interventions.

Author	Type of Intervention	Length of the intervention and follow up period	Intervention details	Background of the trainers	Tools of Evaluation
Singhal, *et al.* 2014 [26]	School based intervention	Intervention: 1 month Follow up- After one month	*Intervention group:* Coping skills program with eight weeks of interactive session's on-identifying negative thinking, changing to positive thinking, using the ABC challenge negative thoughts, using social detective game to solve relationship problems and dealing with academic stress. *Control group*-One psycho-educatory interactive session	Researchers of the intervention administered the program within the school premises. However, the structure and content of program were validated by experts that included 1 psychiatrist and 1 clinical psychologist, both with 20 years of experience in clinical intervention with adolescents.	-Children's Depression Inventory (CDI) -Centre for Epidemiological Studies Depression Scale for Children (CES-DC) -Children's Automatic Thoughts Scale (CATS) -Cognitive Triad Inventory for Children (CTI-C) -Scale for Assessing Academic Stress (SAAS) -Adolescent Coping Orientation to Problems Experienced Inventory (A-COPE) -Social Problem Solving Inventory-Revised-Short Form (SPSI-R) Pre and Post feedback questionnaire
Sarkar, *et al.* 2017 [27]	School based intervention	Intervention 2 years Pre-post-test And follow up- After 3 months Follow up-None	Life skills education-health empowerment intervention using modified National Institute of mental health and neuro sciences (NIMHANS) module of intervention	The training was administered K.S the first author of this study who was supervised by two experts A.D and M.P.S who are co-authors of this study.	-Child Youth Resilience Measurement scale. -Self Determination Scale

Srikala, *et al*. 2010 [28]	School based intervention	Intervention-1 year<br>Pre-post (Case control)	Mental health promotion among adolescents in schools using life skills education (LSE) and teachers as life skill educators using the NIMHANS Model	Teachers were trained by the master trainers as life skills educators.	-Preadolescent Adjustment Scale (PAAS)<br>-Generalized Self-Efficacy Scale (GSES)<br>-Strengths and Difficulties Questionnaire<br>-Class Room Indicators
Das, *et al*. 2010 [29]	School based intervention	Intervention-2 months<br>Pre-post test<br>Follow up-None	Health education intervention with multiple sessions for two months community medicine professionals and followed by clinical examination by Obstetrics and gynaecology (OBG) specialists. Free referral services and medication was also given to those in need.	Health education intervention was delivered by community medicine professionals and examined by CM and OBG personnel's	Close-ended Pre- and Post-test questionnaires on knowledge and attitude
Leventhal, *et al*. 2015 [30]	School based intervention	Intervention- 5 months<br>Pre-post (case control)	This study combined girl's resilience curriculum (psychosocial assets and well-being) with physical health curriculum to see the effect of the combined resilience and physical health program.	51 people with bachelors and below education, were trained by 4 master trainers with master level above education and experience in training or management in a related field	-Schwarzer's General Self-Efficacy Scale<br>-Patient Health Questionnaire (PHQ-9)<br>-General Anxiety Disorder (GAD-7)<br>-Kidscreen -52 Psychological Wellbeing Subscale<br>- Kidscreen -52 Social Support and Peers Subscale
Shinde, *et al*.2020 [31]	School based intervention	Intervention – 17 months<br>Baseline (2015) Follow after 8 months (T1) and then at 17 months (T2)<br>Case control	Strengthening Evidence base on school-based interventions for promoting adolescent health (SEHER) is a multicomponent intervention that had three arms and a control group.<br>1. The whole school intervention activities included: school health	Teacher SM and SM underwent a same curriculum training for one week followed by in-service training through separate monthly meetings	Beyond Blue School Climate Questionnaire (BBSCQ)

			promotion committee, awareness generation, speak up box, wall magazine.), competition between the two intervention groups such as quizzes, sports, etc. and health policies<br>2. Group activities: peer group meetings and workshops<br>3. Individual activities: problem-solving-based counselling for both the groups	-6 supervisors with a master's degree in psychology, sociology or social work were trained for a week to supervise the TSM and SMs	
Singhal, *et al.* 2018 [32]	School based intervention	Intervention- 2 years<br>Pre-post and follow up at 3 months<br>Case Control	The intervention group received eight weekly coping skills programs with same gender groups (4-8 adolescents each), and the control group adolescents received one interactive psycho-educatory session.<br>.	The intervention was administered by the authors of the study within the school premises.	-Beck's Depression Inventory (BDI)<br>-Children's Depression Inventory (CDI)<br>Centre for Epidemiological Studies Depression Scale for Children
Michelson, *et al.* 2019 [33]	School based intervention	Pilot design- 2 and a half years then follow up modifications in the pilot design	1) Problem-focused coping was addressed using a guided self-help modality, delivered through an illustrated workbook with character based vignettes 2) Emotion-focused coping skills was introduced in supplementary hand outs<br>3) Self-help materials to be supported by counsellor guidance were delivered through face-to-face contacts<br>4) Parents were not ordinarily involved in sessions	*Initial design*-Lay counsellors<br>*Modified Pilot 1*-Therapists (Female psychologists with postgraduate degree, 3 counselling assistants<br>*Modified Pilot 2*- Therapists-newly recruited counsellors including 9 college graduates (both male and female) with no prior training in psychotherapy	Session-by-session assessments

Azeez A, 2015 [34]	School based intervention	Intervention- 28 hours (7 sessions, 4 hours each)<br>Pre-post test	The intervention package includes seven sessions with 10 core areas of life skills education with emphasis on psychological well-being and self-esteem.<br>The training comprised of ice-breaks, role plays, games, group discussions and relaxation techniques.	School counsellors	-Ryff's Scale of Psychological Well Being (RSPWB-22)<br><br>-Culture free Self Esteem Inventory (40 items)
Balaji, *et al*. 2011 [35]	Community Intervention	Intervention- 18 months<br>Pre-post survey	1. Peer education program delivered by youth peer leaders supported by the community advisory board in the rural setup and by trained teachers and student forums in the urban educational setups<br>2. Teachers training program: teachers in educational institutes were trained on effective teaching methods, strategies to improve teacher-student relationships, detection, and management of common health problems faced by youth in school settings, and counselling skills.<br>3. Distribution of health information material.	-Trained Peer leaders in delivered in the rural set up Trained teachers and student forums in urban set ups. They were trained by psychologists and social workers experienced in the field of adolescent health.	-General Health Questionnaire (GHQ-12)<br>-In-depth interviews with youth, parents, teachers, and heads of institutions
Chandra, *et al*. 2014 [36]	Digital intervention	Intervention-1 month<br>Feedback was collected after 1 month	The adolescents received a message every day for a month. Messages alternated between positive mental health tips or helpline information. The helpline message asked them to message or call back if they felt like talking to someone when emotionally upset.	Digital messages were sent and received back only by researchers. However, content on wellbeing were sought from two mental health experts and two young women from the community.	-Feedback sessions
